# Cervical extension of the thymus mimicking metastatic recurrence of Ewing sarcoma on PET/CT

**DOI:** 10.4103/0972-3919.78259

**Published:** 2010

**Authors:** Sumeet G Dua, Nilendu C Purandare, Sneha Shah, Riddhika Maitra, Venkatesh Rangarajan

**Affiliations:** Bio-Imaging Unit, Tata Memorial Hospital, Parel, Mumbai, India

**Keywords:** Thymic hyperplasia, cervical extension of thymus, PET/CT

## Abstract

Occasionally the thymus may extend in the neck, from its normal location in the anterior mediastinum. The cervical extension, especially when the native thymus is hyperplastic, can mimic a mass. We describe the detection of cervical extension of the hyperplastic thymus, presenting as a suspicious recurrent soft tissue mass in the neck in a patient with Ewing’s sarcoma.

## INTRODUCTION

Cervical extension of the thymus is often mistaken for a soft tissue mass in the neck, particularly in children and young adults. The problem in differentiating thymic tissue from a mass is compounded when the thymus is hyperplastic and shows increased fluorodeoxyglucose uptake. We describe the case of a child on follow-up for Ewing sarcoma in whom we detected an avid thymic extension in the neck, which mimicked recurrent disease both anatomically and functionally.

## CASE REPORT

**Figure 1:**
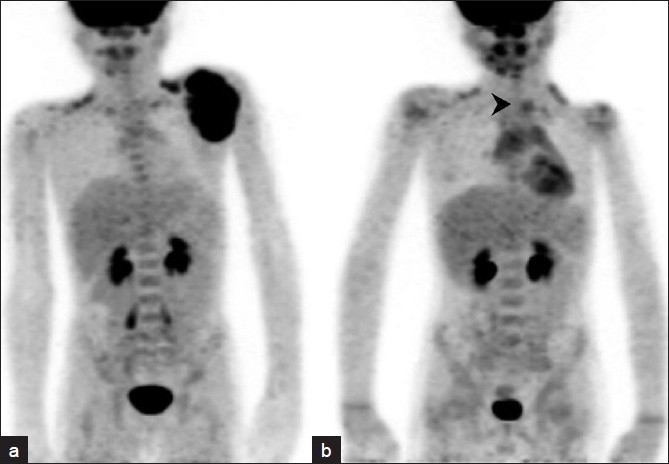
Pre and post treatment maximum intensity projection images

An 8-year-old child presented in 2009 with a swelling on the left side of the back in the scapular region. Clinical examination revealed a lobulated nontender soft tissue mass. Biopsy was suggestive of Ewing sarcoma. A staging positron emission tomography/computed tomography (PET/CT) study performed at that time revealed increased tracer accumulation in the soft tissue mass associated with the left scapula [Figure 1a]. The rest of the study was unremarkable. There were no lung nodules to suggest metastatic dissemination. Subsequently, the patient received chemotherapy and underwent scapulectomy. A year after treatment he was asymptomatic and clinical examination was unremarkable. He was then referred to us for a follow-up PET/CT. This showed a focus of mildly increased uptake in the left lower neck [arrowhead in Figure 1b], in addition to minimal treatment-related hypermetabolism at the postoperative site. There were no other foci of abnormal uptake.

**Figure 2:**
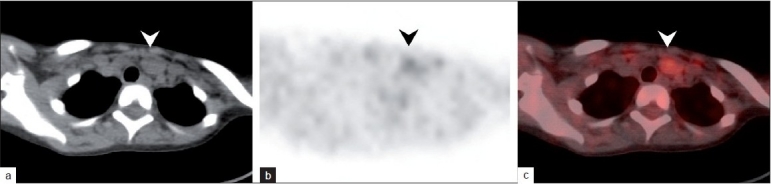
Axial CT, PET and fusion images from the follow up PET/CT

The focus of uptake in the neck [arrowhead in Figure 2b] localized to well-defined, 2-cm sized lobulated soft tissue [arrowhead in Figure 2a] mass inferior to the thyroid gland on the fusion PET/CT image [arrowhead in Figure 2c]. In view of the scan findings, metastatic recurrence of the tumor in the neck was suspected. The uptake seen on the PET images however was low (maximum standardized uptake value 1.9) and an ultrasound-guided cytological examination was suggested to confirm the PET findings. However, the ultrasound examination was unremarkable and failed to show the lesion.

**Figure 3:**
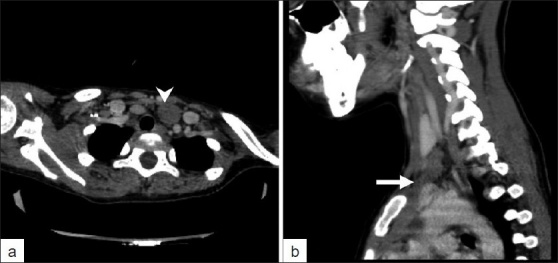
Axial and sagittal contrast enhanced CT images

A dedicated contrast-enhanced CT was then performed to characterize the suspicious lesion reported on the PET/CT study. Axial CT image confirmed the presence of a hypoenhancing lobulated soft tissue lesion between the trachea and the jugular vein [Figure 3a]. Closer inspection of the sagittal reformatted CT images revealed that the suspected soft tissue mass was in fact in anatomic continuity with the thymic tissue in the mediastinum [arrow in Figure 3b] and showed the same density. In the light of all these findings, we arrived at a diagnosis of cervical extension of the thymus.

## DISCUSSION

The thymus gland develops from the third pharyngeal pouch on each side. In the eighth week of intrauterine life the primordial thymus from both sides fuses in the midline and migrates inferiorly to its normal retrosternal location in the superior mediastinum.[1] The migration is sometimes incomplete and an ectopic thymus may be seen in the neck due to arrest in the descent of the gland. More frequently, however, thymic tissue may be seen in the neck as an extension of the native thymus. In fact a recent study identified cervical extension of the thymus in about two-third of children and young adults.[2] On FDG PET the normal thymus shows homogeneous and diffuse uptake and has an inverted V-shape on coronal views.[3] The physiologic uptake gradually disappears with involution of the thymus in adolescence.[4] Variations in the anatomic and metabolic characteristics of the thymus are frequent and it can sometimes mimic a neck or mediastinal mass, which can be a pitfall in the interpretation of imaging studies.[5] In a subset of oncology patients, particularly children receiving chemotherapy, the thymus undergoes hyperplasia and shows increased FDG uptake, which can occasionally be intense and simulate a mass.[4] The situation can be complicated further when increased uptake is seen in the ectopic or cervical thymus. Our patient had a history of receiving chemotherapy, which would account for the increased uptake in the cervical extension of the thymus. The imaging findings were further confounded by the hyperplasia of the cervical extension of the thymus, which gave it the appearance of a mass on unenhanced images. Also the hypermetabolism seen in the cervical thymic extension was on the same side as the primary, which made us think of a recurrent soft tissue mass, an impression further supported by the incongruity of the uptake with that of the native thymus.

It has been shown that cervical extension of the thymus, generally, is anterior to the brachiocephalic vein; has a density identical to that of the thymus, with no intervening fat planes; and does not produce any mass effect on the surrounding structures.[6] All of the above criteria were fulfilled in our case and, accordingly, a diagnosis of cervical extension of the thymus was arrived at and the possibility of metastatic recurrence ruled out.

In summary, a soft tissue mass in the lower neck near the midline, particularly in pediatric patients with a history of chemotherapy, should be viewed with caution. Careful scrutiny of the multiplanar CT reformations and knowledge of the imaging attributes of cervical extension of the thymus can, as shown in our case, thus help the nuclear medicine physician to differentiate it from an actual mass or an enlarged node.
